# ATXN2 and Its Neighbouring Gene SH2B3 Are Associated with Increased ALS Risk in the Turkish Population

**DOI:** 10.1371/journal.pone.0042956

**Published:** 2012-08-20

**Authors:** Suna Lahut, Özgür Ömür, Özgün Uyan, Zeynep Sena Ağım, Aslihan Özoğuz, Yeşim Parman, Feza Deymeer, Piraye Oflazer, Filiz Koç, Hilmi Özçelik, Georg Auburger, A. Nazlı Başak

**Affiliations:** 1 Boğaziçi University, Molecular Biology and Genetics Department, Neurodegeneration Research Laboratory, Istanbul, Turkey; 2 Istanbul University, Istanbul Medical School, Neurology Department, Istanbul, Turkey; 3 Çukurova University, Medical School, Neurology Department, Adana, Turkey; 4 University of Toronto, Samuel Lunenfeld Research Institute, Mount Sinai Hospital, Department of Laboratory Medicine and Pathobiology, Toronto, Ontario, Canada; 5 Goethe University, Experimental Neurology, Frankfurt am Main, Germany; Institut Jacques Monod, France

## Abstract

Expansions of the polyglutamine (polyQ) domain (≥34) in Ataxin-2 (ATXN2) are the primary cause of spinocerebellar ataxia type 2 (SCA2). Recent studies reported that intermediate-length (27–33) expansions increase the risk of Amyotrophic Lateral Sclerosis (ALS) in 1–4% of cases in diverse populations. This study investigates the Turkish population with respect to ALS risk, genotyping 158 sporadic, 78 familial patients and 420 neurologically healthy controls. We re-assessed the effect of ATXN2 expansions and extended the analysis for the first time to cover the ATXN2 locus with 18 Single Nucleotide Polymorphisms (SNPs) and their haplotypes. In accordance with other studies, our results confirmed that 31–32 polyQ repeats in the ATXN2 gene are associated with risk of developing ALS in 1.7% of the Turkish ALS cohort (p = 0.0172). Additionally, a significant association of a 136 kb haplotype block across the ATXN2 and SH2B3 genes was found in 19.4% of a subset of our ALS cohort and in 10.1% of the controls (p = 0.0057, OR: 2.23). ATXN2 and SH2B3 encode proteins that both interact with growth receptor tyrosine kinases. Our novel observations suggest that genotyping of SNPs at this locus may be useful for the study of ALS risk in a high percentage of individuals and that ATXN2 and SH2B3 variants may interact in modulating the disease pathway.

## Introduction

ALS is a late-onset, rapidly progressive and devastating neurodegenerative disorder, which is generally associated with selective degeneration of both upper and lower motor neurons (MNs) in the brain, brainstem and spinal cord. Ten per cent of all ALS cases are inherited and referred to as familial ALS (fALS); the remaining 90% are sporadic (sALS) [1]. Although the mechanisms causing ALS are not well understood yet, several genes have been linked to the disease. Among these, SOD1 involved in oxidative stress, TARDBP and FUS implied in RNA-processing [2], [3], [4], [5], [6], [7], and the two recently identified UBQLN2 and C9ORF72 genes are the most prominent causes of fALS [8], [9], [10]. Recently, genome wide association studies (GWAS) have identified SNPs that are associated with sALS, underscoring the importance of investigating common genetic variations [11], [12], [13].

In addition to rare mutations and common SNPs, a recent publication reported that ATXN2 dysfunction influences the TDP-43-dependent toxicity seen in ALS and that the intermediate-length expansions to 27–33 triplets in the ATXN2 polyglutamine (polyQ) region act as ALS risk factors in 4.7% of North American patients [14]. All follow-up studies confirmed the association of the ATXN2 polyQ expansions with ALS risk in various ethnic populations, including North Americans, Europeans, French-Canadians and Chinese [14], [15], [16], [17], [18], [19], [20]. Further, North-American studies investigated the specificity of the association between ATXN2 and ALS. Ross *et al.* demonstrated that intermediate-length polyQ expansions are associated with neither Alzheimer’s nor idiopathic Parkinson’s diseases, but with ALS and the Parkinson-plus entity progressive supranuclear palsy [19]. In addition, Lee *et al.* as well as Gispert *et al.* reported that among several other polyQ neurodegenerative disease proteins, only ATXN2 is associated with ALS risk [21], [22], suggesting that the physiological functions of ataxin-2 in RNA processing and/or receptor tyrosine kinase endocytosis are relevant [23], [24], [25], [26], [27]. The polyQ expansion is thought to convey a gain-of-function (GOF) effect on the ataxin-2 protein and to provoke insolubility and aggregation of ataxin-2 with its interacting proteins [28], [29], [30]. However, a loss-of-function of ataxin-2, in addition to its GOF, cannot be excluded, since both mechanisms seem to be important modulators of disease manifestation in several neurodegenerative diseases [31].

ATXN2 usually contains a repeat structure with 22 or 23 triplets coding for glutamine and the (CAG)_8_CAA(CAG)_4_CAA(CAG)_8_ sequence; expansion of this domain to a size ≥34 triplets with a pure CAG sequence primarily causes autosomal dominant SCA2 [32], while ATXN2 expansions with CAA interruptions were observed as the cause of Levo-dopa responsive Parkinson’s disease [33]. ATXN2 expansions associated with ALS were reported by Corrado *et al.* to be interrupted by at least one CAA triplet [16], Yu *et al.* identified ATXN2 expansions in 40 ALS patients to be always interrupted by CAA triplets, and defined a haplotype of two ATXN2 SNPs (rs695871 and rs695872) in common between most cases with 3 CAA interruptions and another haplotype in common between most cases with 1–3 CAA interruptions [34], [35].

This study now aims to investigate the association of the ATXN2 chromosomal region with ALS risk in the Turkish population, considering not only the polyQ repeats, but also common SNPs and haplotype patterns.

## Materials and Methods

### Ethics Statement

The Ethics Committee of Boğaziçi University approved the use of patient samples for this study. Written informed consent forms were obtained from all patients. Control samples were collected anonymously.

### PolyQ Expansion Analyses

A total of 236 Turkish ALS patients (158 sALS and 78 fALS) matching El Escorial Criteria [36] were referred to our center from several hospitals throughout Turkey. Fifteen of these patients had already a defined mutation in one of the genes responsible for ALS and these were also included in the study, in addition to 420 Turkish healthy controls without any known history of neurological disorders. These control samples were collected from the Microbiology Department of Haydarpaşa State Hospital in Istanbul. The mean ages of onset of sALS and fALS patients were 48.9 (range: 24–79) and 34.3 (range: 8–80) years, respectively. The average age of the control group was 63.8 (range: 38–97). Male to female ratios were 3∶2 for sALS, 1∶1 for fALS and 1∶1 for controls. DNA was extracted from peripheral blood cells of patients and controls, using the MagNAPure Compact (Roche) DNA isolation systems.

The ATXN2 triplet repeat was amplified from DNA samples of patients and healthy controls, using polymerase chain reaction (PCR) with the forward primer 5′- GGG CCC CTC ACC ATG TCG -3′ and the FAM labeled reverse primer 5′−/56-FAM/CGG GCT TGC GGA CAT TGG -3′. PCR cycles included 5 min. at 95°C, 30 cycles (1 min. at 95°C, 1 min. at 55.7°C, 1 min. and 30 sec. at 72°C) and 5 min. at 72°C. Repeat sizes were determined by GeneScan Analysis (Macrogen Inc., Seoul, Korea) and evaluated independently by two authors (SL and ÖÖ). The reproducibility of the GeneScan analyses was validated via repeating 25 samples among 236 ALS cases and 45 samples out of 420 controls, corresponding to ∼10% of both cohorts. A SCA2 positive individual with 41 ATXN2 repeat expansion size was used as an internal control in both PCR amplifications and GeneScan analyses. ALS patients with an ATXN2 expansion were further subjected to DNA sequencing, to assess the presence of CAA interruptions (RefGen Inc, Ankara, Turkey).

Fisher’s exact test was applied to evaluate the genetic association of ATXN2 expansion sizes with ALS risk, under both the allelic and genotypic models.

### SNP and Haplotype Association Analyses

In our independent GWA study (unpublished data) performed earlier, we investigated 733,202 SNPs in 116 out of the above 158 Turkish sALS patients and 109 age- and sex- matched neurologically healthy individuals, using the Illumina HumanOmniExpress SNP array. To examine the association of ATXN2 locus variants with ALS risk in the Turkish cohort under study, we extracted 250 kb genotype data, comprising the ATXN2 locus and the surrounding 50 kb (25 kb from 5′ and 3′ ends) using PLINK software (http://pngu.mgh.harvard.edu/purcell/plink/) [37]. The SNPs with a Hardy-Weinberg equilibrium (HWE) of >0.05 and a minor allele frequency (MAF) of >0.01 were included. Haploview 4.2 software was also used to visualize the haplotype blocks in the ATXN2 and the neighbouring SH2B3 genes (http://www.broad.mit.edu/haploview/haploview) [38]. The haplotype blocks, including SNPs in linkage disequilibrium (LD), were determined via the Gabriel method (CI: 0.98–0.7) [39]. We performed 1000 permutation tests to generate empirical p-values.

## Results

### ATXN2 Expansion Analyses

**Figure 1:**
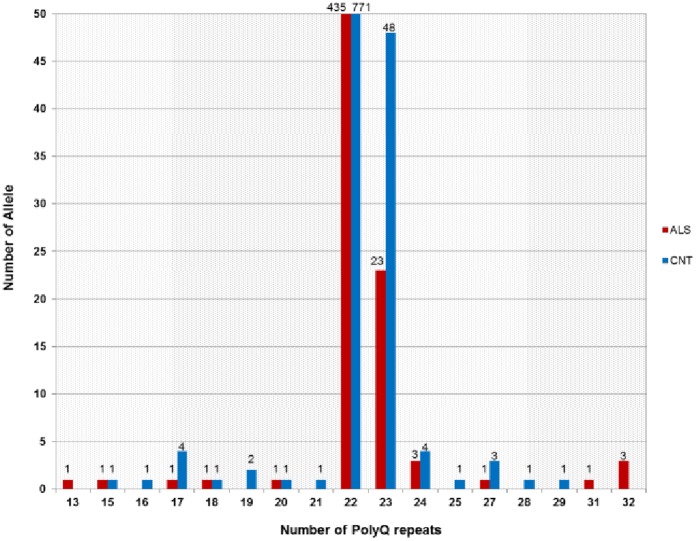
Distribution of ATXN2 repeat sizes, represented as polyQ triplet numbers. Turkish ALS patients are represented in red and healthy controls in blue bars; numbers of the individuals having the relevant alleles are shown on the top of each bar.

We investigated the ATXN2 expansion sizes of 236 ALS patients and 420 healthy controls from Turkey, using GeneScan analysis. The lengths of the ATXN2 repeat alleles varied from 13 to 32 units in ALS patients and from 15 to 29 units in controls (Figure 1). The commonly found 22 repeat allele accounted for approximately 92% of both ALS and control populations. None of the 15 ALS patients, carrying various mutations in different ALS disease genes (SOD1, FUS, UBQLN2, OPTN, SPG11 and PLEKHG5) showed expansions in ATXN2 (p = 0.78) (Table S1). In the allelic model, the MAF of the 31 allele was 0.21% (1/472 alleles) in cases, whereas this allele was absent in controls. The MAF of the 32 allele was 0.63% (3/472 alleles) in cases and again this allele was absent in controls. Fisher’s exact test detected a significant difference for alleles coding a polyQ size >30 between cases and controls (p = 0.016). Also in the genotypic model, the heterozygous presence of a polyQ size >30 showed statistically significant association with ALS risk according to Fisher’s exact test (p = 0.0172) (Table 1). Sequencing of the ATXN2 expansions of the four ALS patients with sizes 31 and 32 showed that three patients had a single CAA interruption within the CAG repeat, while the remaining patient has a pure CAG tract. Clinical information and the sequence data of these four ALS patients are shown in Table 2.

**Table 1:** The frequency of ATXN2 expansion sizes in ALS and controls (in the genotypic model).

	≤30 repeats	>30 repeats	*p*-value^a^
**ALS (n = 236)**	232 (98.3%)	4 (1.69%)	0.01721
**Controls (n = 420)**	420 (100%)	0	

a Fisher’s exact test.

**Table 2:** Clinical characteristics of four Turkish ALS patients with ATXN2 expansions.

ALS No	Gender	Birth	AO	AD	Site of Onset	Genotype	Sequence Composition
**sALS20** *	female	1950	52	57	LE	22/31	(CAG)_21_CAA(CAG)_9_
**sALS39**	male	1962	39	44	LE	22/32	(CAG)_32_
**sALS180** *	female	1929	77	alive	Bulbar	22/32	(CAG)_22_CAA(CAG)_8_
**fALS304**	female	1982	8	alive	Bulbar, LE	23/32	(CAG)_23_CAA(CAG)_8_

AO: age of onset, AD: age at death. UE: upper extremity, LE: lower extremity, ULE: upper and lower extremity.

* GWA- genotyped.

### ATXN2 Locus SNP and Haplotype Association Analyses

**Figure 2:**
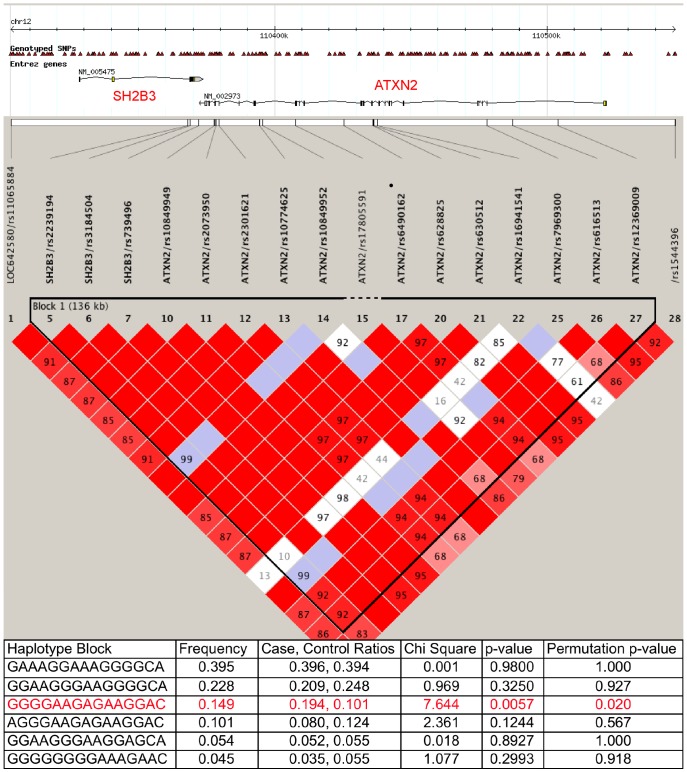
Haplotype block analysis of ATXN2 locus on chromosome 12q (110,300,000–110,550,000). Genotyped SNPs located in 250 kb region were analyzed by Haploview 4.2 in order to identify haplotype blocks with ALS risk. A 136 kb large haplotype block, highlighted in red, was observed in the analysis. The frequencies of haplotypes defined in this haplotype block, the p-values and permutation p-values are shown.

We investigated a 250 kb region on chromosome 12q in 116 out of the above 158 Turkish sALS patients, including two of the four ALS patients with ATXN2 expansions. Ten of 28 SNPs were excluded due to low HWE and MAF scores. In single marker analysis, none of the SNPs within the ATXN2 gene by itself showed any significant association with ALS risk, but a trend towards association was observed for the SNP rs2239194 within the SH2B3 gene (p = 0.063) (Table S2). On the other hand, haplotype analysis of the region, using the Haploview program, demonstrated a strong association for a 136 kb 15-SNP haplotype block (including rs2239194), which contains both the ATXN2 and SH2B3 loci. One haplotype (GGGGAAGAGAAGGAC, MAF = 0.149, F_cases_ = 0.194, F_controls_ = 0.101) had a significant *p*-value of 0.0057 and correlated with an increased ALS risk (OR: 2.23). This risk haplotype was observed in heterozygous state in both Turkish ALS patients with ATXN2 expansions, which were part of the GWA genotyping study. Using permutation analysis, which eliminates false positive data after multiple testing more effectively than Bonferroni corrections [40], this haplotype-risk association retained its statistical significance (p = 0.02) after 1000 permutations (Figure 2).

## Discussion

The first analysis of Turkish ALS patients regarding ATXN2 confirms its role as a risk factor. More importantly, this study identifies a common risk haplotype for ALS, containing the ATXN2 and its neighbouring SH2B3 gene.

The initial observation that ataxin-2 acts as a modifier protein of TDP-43 overexpression toxicity as a model of ALS risk was consistent from *Saccharomyces cerevisiae* via *Drosophila melanogaster* to *Homo sapiens* [14], and the role of ATXN2 intermediate expansions as ALS risk factor was reproduced in every follow-up study [15], [16], [17], [18], [19], [20], [21], [22], [34], [41], [42]. This effect is now also validated in the Turkish population. We did not observe a specific geographic distribution within Turkey or a particular phenotype effect. The effect was limited to the expansion sizes 31 and 32 in this study, while the association was not significant for intermediate expansion alleles of size <30, and the very rare occurrence of large expansion alleles in ALS was not detected, as would be expected from the limited number of patients available. CAA interruptions were detected in the expanded alleles of three out of four ALS patients, but a pure CAG expansion was detected in one ALS patient, suggesting that CAA interruptions are not a prerequisite for ALS manifestation.

In our study, novel evidence indicates that ALS risk is impacted by a 15-SNP haplotype block in linkage disequilibrium across the genes ATXN2 and its downstream neighbour SH2B3. Haplotype association became more significant when SNPs from both the ATXN2 and SH2B3 genes were included; this suggests a role of the SH2B3 gene in ALS risk. The SH2B3 protein, also known as LNK, is a member of the SH2B (1–3) adaptor protein family. They all contain Src Homology 2 (SH2) domains, pleckstrin motifs and proline-rich regions. Thus, they can bind to phosphatidylinositol-lipid containing membranes and to the phosphorylated tyrosine residues, e.g. of receptor tyrosine kinases, modulating the signal transduction that controls proliferation and growth. They exert strong effects in hematopoiesis [43], [44], [45], [46]. Preliminary studies in the nervous system indicate that SH2B3, expressed in cortical neurons from embryonic stages, competes with the other family members (SH2B1 and SH2B2) and inhibits the NGF-induced differentiation of PC12 cells reducing the neurite outgrowth of cortical neurons, through binding of its SH2 domain to the NGF receptor and repressing the PI3K pathway [47]. Previously, a SNP (rs3184504) in the SH2B3 gene was found associated with multiple sclerosis [48]. ATXN2 has similar features with SH2B3 since it contains proline-rich regions that interact with Src Homology 3 domains, it associates with receptor tyrosine kinases and modulates the signaling control of growth [23], [27].

This first analysis of Turkish ALS patients on ATXN2, not only confirmed its role as a risk factor in rare cases with intermediate polyQ expansions, but also revealed novel evidence that SNPs across the ATXN2/SH2B3 genomic locus may modulate risk in a substantial fraction of ALS patients. These data need to be validated in large and independent populations. In the light of these findings, our results implicate a genetic (and a possible biological) interaction between ATXN2 and SH2B3 genes, therefore we propose that it will be useful to investigate genetic variations in this genomic region of ALS patients.

## Supporting Information

Table S1 **Data on Turkish ALS patients with previously identified mutations.**
(DOC)

Table S2 **Association Analysis of 18 SNPs across 250 kb at the ATXN2 Locus.**
(DOC)
