# Supplementary material for: ATXN2 and Its Neighbouring Gene SH2B3 Are Associated with Increased ALS Risk in the Turkish Population
Source: PLoS One. 2012 Aug 20;7(8):e42956. doi: 10.1371/journal.pone.0042956 (PMC3423429; doi:10.1371/journal.pone.0042956)
Supplement: Table S1 — Data on Turkish ALS patients with previously identified mutations. (DOC) [file pone.0042956.s001.doc]

**Table S1. Data on Turkish ALS patients with previously**

**identified mutations.**

| **Patient No** | **Gender** | **Age of onset** | **Mode of inheritance of the patients** | **Mutation** | **ATXN2 PolyQ repeat** | **Site of onset** |
| --- | --- | --- | --- | --- | --- | --- |
| **ALS 61** | female | 53 | AD | SOD1: L144F | 22/22 | LE |
| **ALS 221** | male | 20 | AD | SOD1: A4S | 22/22 | LE |
| **ALS 226** | male | 19 | AD/IP | SOD1: H71Y | 22/22 | ULE |
| **ALS 147** | male | 50 | AR | SOD1: D90A | 17/22 | LE |
| **ALS 310** | male | 55 | AR | SOD1: D90A | 22/22 | LE |
| **ALS 191** | male | 28 | AR | SOD1: N86S | 22/22 | LE |
| **ALS 264** | male | 16 | IP | FUS: ∆143-148 | 22/22 | LE |
| **ALS 131** | female | 16 | X-linked | UBQLN2: M392I | 22/22 |  |
| **ALS 155** | male | 12 | X-linked | UBQLN2: S340I | 22/22 | ULE |
| **ALS 175** | male | 22 | X-linked | UBQLN2: P525S | 22/22 | UE |
| **ALS 268** | male | 26 | X-linked dom. | UBQLN2: P560S | 22/22 | UE |
| **ALS 350** | male | 14 | X-linked | UBQLN2: M392I | 22/22 | LE |
| **ALS 256** | male | 42 | AR | OPTN: ∆ 2 bp  in 359 aa | 22/22 | LE |
| **ALS 132** | male | 14 | AR | SPG11: F2265L | 22/22 | LE |
| **ALS 167** | male | 13 | AR | PLEKHG5: P630H | 22/22 | LE |

AD: autosomal dominant, AR: autosomal recessive, IP: incomplete penetrance, UE: Upper extremity,

LE: lower extremity, ULE: upper and lower extremity, ∆: deletion, aa: amino acid.

All patients with one exception (ALS 147: 17/22), have ATXN2 polyQ as 22/22 repeats.
