# Supplementary material for: ATXN2 and Its Neighbouring Gene SH2B3 Are Associated with Increased ALS Risk in the Turkish Population
Source: PLoS One. 2012 Aug 20;7(8):e42956. doi: 10.1371/journal.pone.0042956 (PMC3423429; doi:10.1371/journal.pone.0042956)
Supplement: Table S2 — Association Analysis of 18 SNPs across 250 kb at the ATXN2 Locus. (DOC) [file pone.0042956.s002.doc]

**Table S2. Association Analysis of 18 SNPs across 250 kb**

**at the ATXN2 Locus.**

| **#** | **Name (SNP)** | **Associated**  **Allele** | **Case, Control Ratios** | **Chi Square** | **p-value** |
| --- | --- | --- | --- | --- | --- |
| **1** | LOC642580/rs11065884 | G | 0.348, 0.317 | 0.494 | 0.482 |
| **5** | SH2B3/rs2239194 | G | 0.921, 0.867 | 3.457 | 0.063 |
| **6** | SH2B3/rs3184504 | A | 0.396, 0.394 | 0.001 | 0.98 |
| **7** | SH2B3/rs739496 | G | 0.329, 0.298 | 0.49 | 0.4838 |
| **10** | ATXN2/rs10849949 | G | 0.330, 0.298 | 0.54 | 0.4623 |
| **11** | ATXN2/rs2073950 | A | 0.281, 0.239 | 1.03 | 0.3102 |
| **12** | ATXN2/rs2301621 | A | 0.283, 0.239 | 1.127 | 0.2884 |
| **13** | ATXN2/rs10774625 | A | 0.409, 0.399 | 0.043 | 0.8358 |
| **14** | ATXN2/rs10849952 | A | 0.952, 0.940 | 0.308 | 0.5791 |
| **15** | ATXN2/rs17805591 | G | 0.969, 0.959 | 0.361 | 0.548 |
| **17** | ATXN2/rs6490162 | G | 0.281, 0.239 | 1.03 | 0.3102 |
| **20** | ATXN2/rs628825 | A | 0.319, 0.294 | 0.327 | 0.5677 |
| **21** | ATXN2/rs630512 | A | 0.322, 0.294 | 0.416 | 0.5187 |
| **22** | ATXN2/rs16941541 | G | 0.948, 0.945 | 0.018 | 0.8927 |
| **25** | ATXN2/rs7969300 | G | 0.952, 0.940 | 0.308 | 0.5791 |
| **26** | ATXN2/rs616513 | A | 0.322, 0.284 | 0.738 | 0.3903 |
| **27** | ATXN2/rs12369009 | C | 0.307, 0.280 | 0.397 | 0.5284 |
| **28** | /rs1544396 | A | 0.778, 0.743 | 0.76 | 0.3832 |
